# Data-fusion for in-situ monitoring and molten state identification during LPBF of NiCoCr medium-entropy alloy

**DOI:** 10.1038/s41598-024-65545-9

**Published:** 2024-06-26

**Authors:** Hong Li, Shaohua Yan, Yu Fu

**Affiliations:** 1 Shenzhen Key Laboratory of Intelligent Optical Measurement and Detection, College of Physics and Optoelectronic Engineering, Shenzhen University, 3688 Nanhai Avenue, Shenzhen, 518060 China; 2 State Key Laboratory of Radio Frequency Heterogeneous Integration, Shenzhen University, 3688 Nanhai Avenue, Shenzhen, 518060 China

**Keywords:** Laser powder bed fusion, Process monitoring, Spatter, Plume, Kalman filter, PSO-XGBoost, Materials science, Optics and photonics, Physics

## Abstract

Laser powder bed fusion (LPBF) is an additive manufacturing technology with high practical value. In order to improve the quality of the fabricated parts, process monitoring has become a crucial solution, offering the potential to ensure manufacturing stability and repeatability. However, a cardinal challenge involves discerning a precise correlation between process characteristics and potential defects. This paper elucidates the integration of an off-axis vision monitoring mechanism via a high-speed camera focused on capturing the single-track melting phenomenon. An innovative image processing method was devised to segment the plume and spatters, while Kalman filter was employed for multi-object tracking of the spatters. The features of both the plume and spatters were extracted, and their relationship with molten states was investigated. Finally, the PSO-XGBoost algorithm was utilized to identify five molten states, achieving an accuracy of 92.16%. The novelty of this approach resides in its unique combination of plume characteristics, spatter features, and computationally efficient machine learning models, which collectively address the challenge of limited field of view prevalent in real production scenarios, thereby enhancing process monitoring efficacy. Relative to existing methodologies, the proposed PSO-XGBoost approach offers heightened accuracy, convenience, and appropriateness for the monitoring of the LPBF process. This work provides an effective and novel approach to monitor the LPBF process and evaluate the part fabrication quality for complex and changeable working conditions.

## Introduction

In recent decades, the advancements in metal additive manufacturing technology have been paramount, rendering the technology indispensable for sectors such as aerospace and medical instrumentation. Laser Powder Bed Fusion (LPBF) emerges as a distinguished and prospective metal printing technology, whereby designated regions of powder layer undergo sequential laser-induced melting, facilitating component fabrication. One of the salient virtues of LPBF is its capability to generate intricate components with excellent mechanical properties. Nonetheless, the quest for achieving stability, reliability, and repeatability in the manufacturing process poses significant impediments^1^. One significant challenge arises from the dynamic nature of the laser-material interaction during the additive manufacturing process. This interaction involves complex and multi-scale phenomena, including melt pool dynamics and rapid solidification, making it challenging to fully understand the building process. Furthermore, the quality of the building parts is highly influenced by various process parameters^2^ and environmental conditions, making it difficult to determine the optimal parameter combinations. Therefore, the manufacturing process is susceptible to the occurrence of diverse defects such as balling, porosity, and cracking^3^, affecting the quality of the products. Therefore, it is particularly important to find an effective means of monitoring.

Presently, the prevailing monitoring techniques for additive manufacturing defects encompass X-ray computed tomography^4^, optical microscopy metallographic observation^5^, electron microscopy observation^6^ and so on. However, the majority of these approaches are implemented after the material has been consumed, rendering them unable to offer timely warnings or formulate suitable counter strategies when defects arise. Furthermore, these monitoring methods are laborious, expensive, and fail to meet the demands of real-time monitoring. Currently, research on online monitoring systems for defects in LPBF is still at an infant stage, lacking commercially available monitoring systems that are both low-cost and highly reliable. At this stage, several monitoring methods have been adopted to investigate online monitoring systems for process quality. These methods primarily focus on monitoring processing signals related to the manufacturing process or quality, such as heat^7^ and acoustics^8^ etc. However, the sensors used in these methods generally need to be installed inside the printing chamber. Additionally, these approaches are overly complex and susceptible to environmental interference.

Apart from the physical quantities mentioned above, optical emissions can also provide a wealth of information about the melt pool such as surface vaporization, plasma radiation, and spatter-related information^9–11^. Plume and spatters occur during the manufacturing process and carry many information, which is valuable for in-situ monitoring. The spatter is caused by the recoil pressure, the Marangoni effect^12^ and vapor-induced entrainment^13,14^. Wang et al.^12^ investigated the mechanisms and characteristics of spatter generation in SLM process and their effects on the properties of printed parts. Zheng et al.^15^ revealing that the occurrence of powder spattering is more closely associated with the stability and evolution of the vapor plume, as well as the resultant melt track formation, rather than the changing of volumetric energy density. In another study, Schwerz et al.^16^ detected redeposited spatters and correlated them with lack of fusion defects, they also found that the spatial distribution of redeposited spatter on the powder bed is related to the scanning strategy employed. While previous research efforts have mostly focused on qualitative analysis, and there is still limited quantitative numerical analysis and application regarding spatters and plumes during LPBF. Dou et al.^17^ attempted to classify different quality states by using spatter signatures and proved effective. However, only three spatter signatures including pixel size, angle and quantity, were investigated due to the limitation of image processing method. Thus, there is still a scarcity of comprehensive quantitative studies on the characterization of plumes and spatters in the LPBF process.

Besides, high-speed cameras are commonly used for monitoring the selective laser melting (SLM) process and provide a visual means of observing the behavior of the melt pool, spatters, and vapor plumes. These cameras capture rapid sequences of images, allowing for a detailed analysis of the dynamic processes occurring during the printing process. There are typically two monitoring methods available: coaxial and off-axis. The coaxial monitoring method requires the camera to be fixed inside the printing chamber, making it susceptible to the environmental condition and limiting observations to the melt pool only. On the other hand, the off-axis monitoring system has the advantages of simple installation and flexible monitoring position, free from the effects of the chamber environment, and provides a more comprehensive view of the process. Therefore, we have chosen the off-axis observation method to capture relevant information about plume and spatter behaviors.

At the same time, the burgeoning progression of machine learning (ML) also supports the monitoring of optical emission signals^18,19^. Many studies have introduced machine learning methods for process monitoring based on traditional signal processing and numerical analysis. Repossini et al.^20^ conducted a pioneering study on the feasibility of using spatter-related information and the logistic regression model to characterize the quality of fabricated parts. The results indicate that incorporating spatter as a process characteristic driving factor significantly enhances the capability to detect both under-melting and over-melting conditions. Ye et al.^21^ used the deep belief network algorithm for the potential defects recognition, obtaining the classification rate of 83.40%. While this method eliminates the need for a feature extraction step, it typically requires multiple iterations of unsupervised pre-training and supervised fine-tuning, which leads to longer training times. In a comparative analysis, Zhang et al.^22^ compared two classification methods, Support Vector Machine (SVM) and Convolutional Neural Network (CNN), for quality recognition, with the latter manifesting superior precision at 92.7%. The feasibility of this method has been demonstrated. Although CNNs have the ability to learn complex relationships, too many adjustable parameters in a CNN make it challenging to handle and interpret. Dou et al. utilized a high-speed camera to capture images of hot spatters and analyzed three specific features of these spatters. Additionally, they employed an AdaBoost CART classification model to establish the relationship between the spatter features and the performance of the parts. However, this method only achieved an accuracy of 71.1%. Furthermore, the AdaBoost model demonstrated weak robustness and noise resistance capabilities. Therefore, during in-situ monitoring processes, there is a demand for simple data processing, high identification accuracy, strong noise resistance, and convenient parameter adjustment. The PSO-XGBoost model can meet these requirements for process monitoring.

Chen et.al first introduced the XGBoost model in 2016^23^, which is an integrated learning algorithm based on tree structures. The model utilizes a second-order Taylor expansion to enhance accuracy and incorporates regularization terms to prevent overfitting. However, the XGBoost model involves some parameters, and their configurations can significantly impact the performance of the final model. Empirical parameter tuning often proves challenging in obtaining optimal values, thereby increasing the complexity of the task^24^. Meanwhile, the influence of XGBoost parameters on accuracy is nonlinear, and these parameters often take non-integer values. Conventional optimization algorithms, such as integer linear programming, are not applicable in this context. A commonly employed swarm optimization algorithm known as the particle swarm algorithm^25^ is well-suited to address this uncertainty issue. To assess the defect states more accurately, our study proposes a PSO-XGBoost algorithm model. Both machine learning detection techniques and datasets are very important, and detection becomes difficult if the captured anomaly images are of low resolution^18^. Therefore, it is most appropriate or compatible to use preprocessing methods to ensure the quality of machine learning techniques and datasets^26^. Essentially, machine learning techniques and hybrid models provide better solutions for real-time anomaly detection^27^.

This study aims to investigate the effects of various parameters on spatters, plumes, and melt tracks while also analyzing the underlying mechanisms. The high-speed camera captured the single-track printing process, and plume and spatter features were extracted using image segmentation and multi-object tracking techniques. In addition, the influence of different energy inputs on the molten state was analyzed. Finally, the extracted features were inputted as samples into the PSO-XGBoost model for the classification of the molten states. This work offers an alternative to traditional destructive offline inspection methods for monitoring and evaluating LPBF processes. Moreover, this novel approach has the potential to be extended and applied to other laser processing techniques as well.

## Materials, experimental setup and methods

### Materials and experimental setup

**Figure 1:**
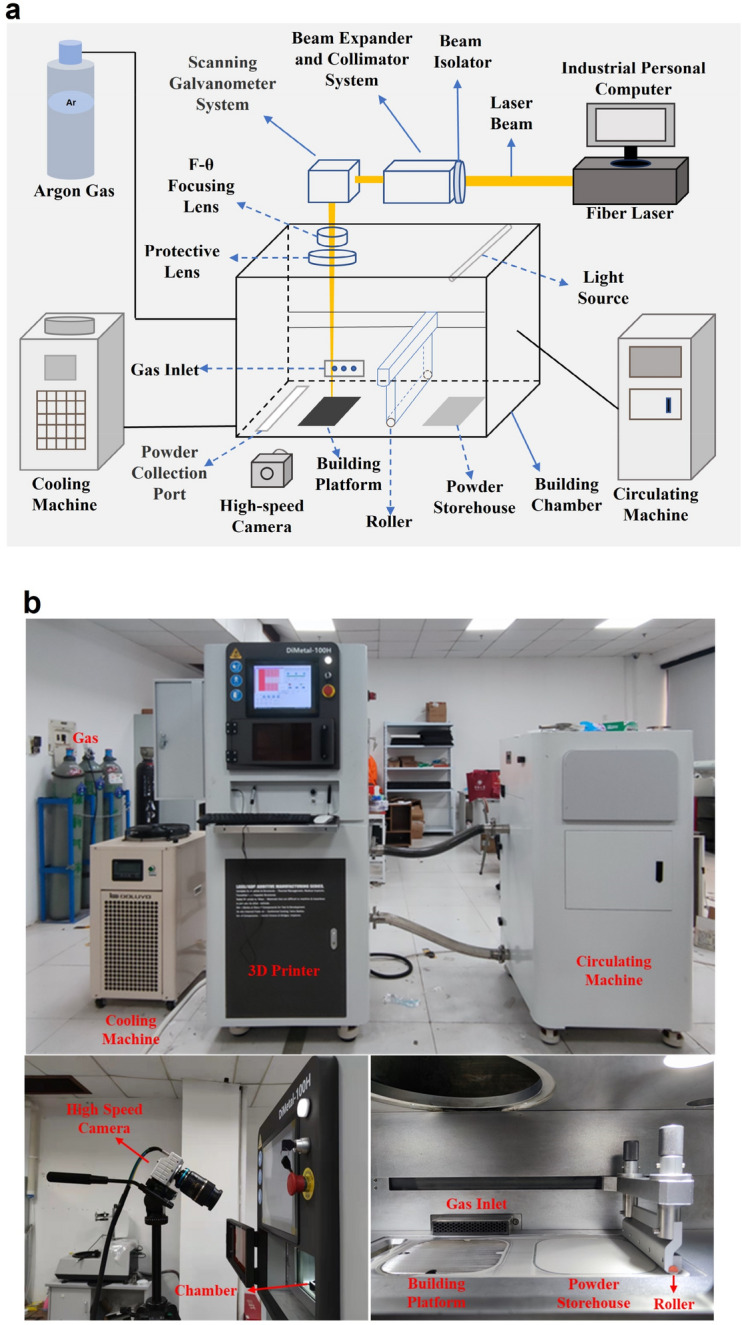
In-situ monitoring system for the LPBF process. (**a**) Schematic of the system. (**b**) Actual scenes of the system.

In multi-principal element alloys (MPEAs), the NiCoCr medium entropy alloy (MEA) stands out for its remarkable strength and ductility, exhibiting superior performance at both low and high operating temperatures^28^. Owing to its exceptional mechanical properties and corrosion resistance, this alloy has garnered significant attention across industries such as aerospace, automotive, and energy, making it also a suitable candidate for applications in metal additive manufacturing^29^. In this study, we employed NiCoCr medium-entropy alloy powder occurring to its with particle sizes ranging from 15 to 53 μm for our experiments. The elemental composition of the powder is detailed in Table 1. Figure 1 illustrates the schematic diagram and the practical setup of the coaxial system used in LPBF. The LPBF device, DiMetal-100H, is equipped with a 200 W fiber laser having a beam diameter of 50–70 μm. The build chamber has dimensions of 100 mm × 100 mm × 100 mm. The camera was positioned outside the printing chamber at a 35° angle relative to the optical axis of the build platform. Simultaneously, the camera's frame rate was configured at 2100 fps with a resolution of 1280 × 720, and the shutter speed was adjusted to 25 μs to minimize motion blur. Various single tracks were obtained by altering the process parameters (as detailed in Table 2), with each track measuring 70 mm in length and following a horizontal scanning direction. Prior to each experiment, the chamber door was sealed, and Argon gas was introduced to evacuate any air, guaranteeing an oxygen-free environment inside. In this study, over 5000 frame images were captured for each parameter combination. It is worth noting that, for configurations involving higher speeds, additional experiments were conducted to ensure a sufficient sample size and maintain sample balance for subsequent machine learning analysis. In the examination of a single track, our primary emphasis was on understanding how various laser energy inputs affect process characteristics and molten states. We did not delve into the impact of powder layer thickness or laser spot diameter. As a result, our analysis was confined to laser linear energy density^30^, defined as:

1 $${E}_{l}=P/v$$

where P is the laser power, and v is the laser scanning speed.

**Table 1:** Chemical composition of NiCoCr medium-entropy alloy powder.

Element (%)/material	Co	Cr	Ni
NiCoCr medium-entropy alloy	35.03	30.23	Bal

**Table 2:** Process parameters used in this study.

Sample #	Laser power (W)	Scan velocity (mm/s)	Energy input (J/mm)
1	100	250	0.4
2	100	200	0.5
3	100	150	0.67
4	100	100	1
5	50	50	1
6	100	50	2
7	150	50	3
8	200	50	4
9	250	50	5
10	300	50	6

### Melt width measurement and molten state assessment

The morphology of the melt track, both from a top-view and a cross-sectional perspective, was examined using a metallurgical microscope. To observe the cross-section of the melt track, the substrate was first cut into small pieces after single-track printing. Subsequently, these cross-sections underwent a series of polishing steps using 200, 400, 800, 1000, and 1500-grit sandpaper. This was followed by a polishing process using alumina suspension with particle sizes of 1, 0.5, 0.04, and 0.02 μm to achieve a smooth surface finish. Finally, the polished samples were etched using a mixture of 1 g CuSO_4_·5H_2_O, 25 ml HCl, and 1.8 ml H_2_SO_4_. A thorough analysis was carried out on the surfaces of the observed melt tracks under the microscope, which encompassed factors such as continuity, width and height uniformity, and roughness. Various molten states were identified as the resultant categories of interest. Furthermore, measurements of the melt tracks were taken under varying parameters to assess the impact of input energy on both the width and height of the melt tracks.

### The overall experimental process

**Figure 2:**
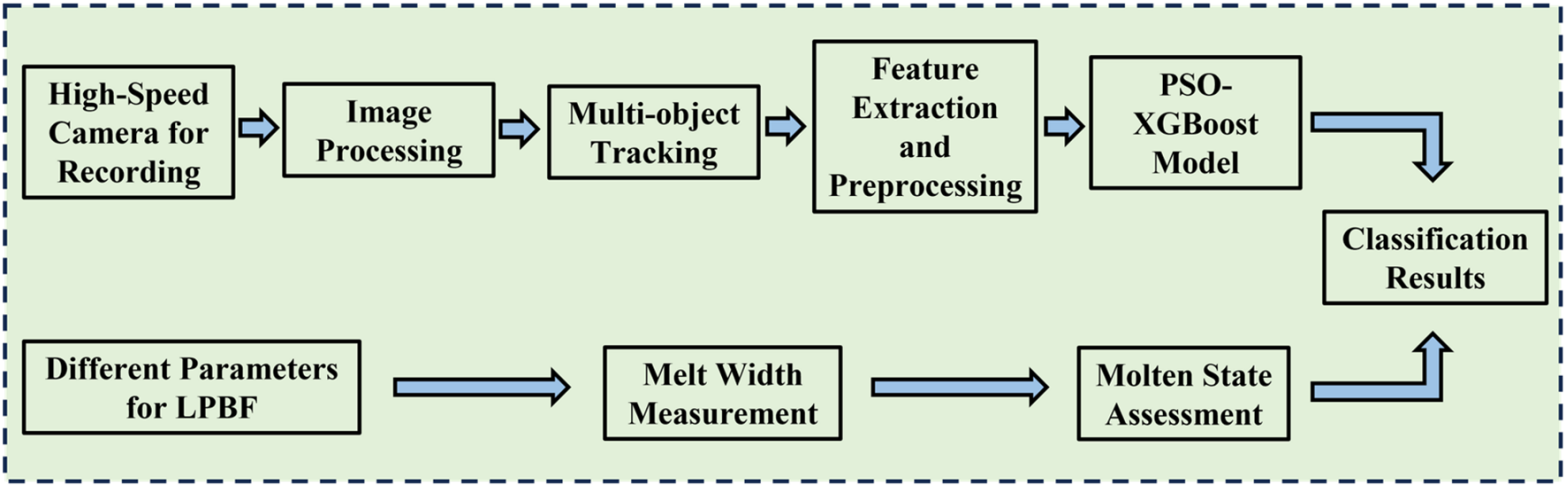
Flow chart of the experimental scheme.

The melting state reflects the defects to some extent and, at the same time, can be used as a basis for classification. Based on the experimental parameters mentioned above, splashing images of the single-track forming process were collected under different linear energy density inputs. The obtained splash process images were segmented to extract features such as average splash and feather flow pixel area, average particle velocity, and number of splashing particles per frame. Based on these features, a thorough analysis and discussion of the interaction mechanism between laser and metal powder were conducted. Finally, in order to validate the accuracy and reliability of the information reflected by the splashing and feather flow image features, a PSO-XGBoost classification model was constructed. The features and results of the single-track experiment were used as inputs and outputs of the model, respectively. The classification accuracy of the model was then tested. The experimental process is shown in Fig. 2.

### Data processing

#### Image processing

**Figure 3:**
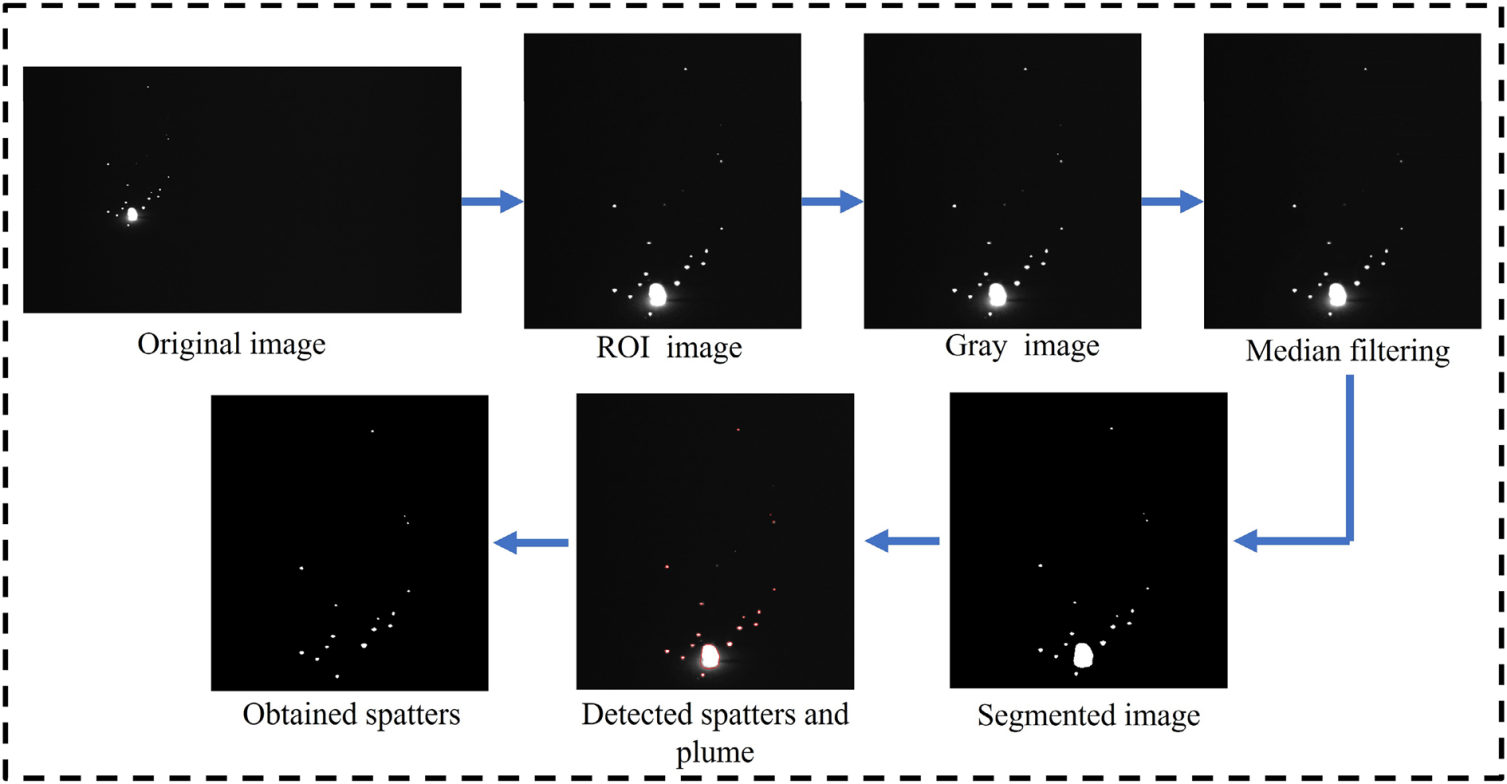
Steps of image processing.

**Figure 4:**
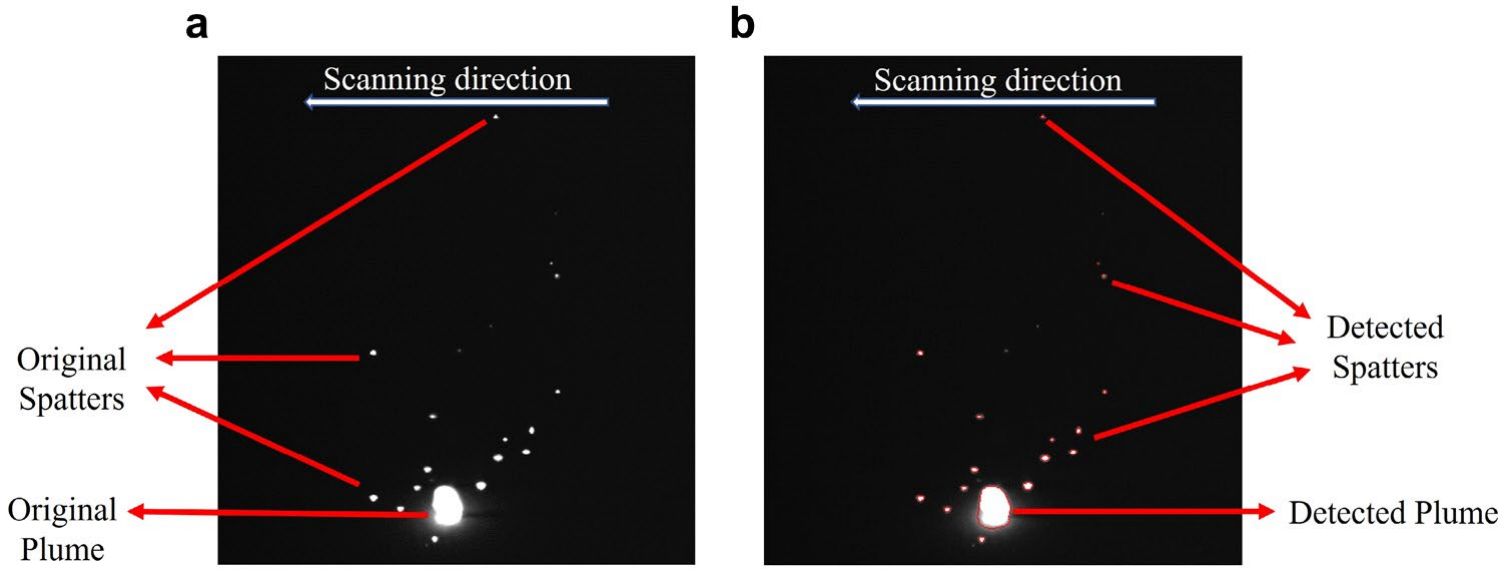
(**a)** The actual spatters and plume. (**b)** The detected spatters and plume.

The original image captured by the camera underwent several processing steps. Firstly, it was cropped to an appropriate size and then converted into a grayscale image. Following this, the resulting grayscale image was subjected to denoising through Median filtering. Subsequently, the Otsu algorithm^31^ was applied to segment the image. This segmentation process effectively differentiates between the plume and spatters due to the substantial disparity in area. For visual reference, Fig. 3 shows the steps of image processing. And the actual and detected plume and spatters can be observed in Fig. 4.

#### Spatter tracking

The Kalman filter is a technique founded on the fundamental concept of minimizing error covariance. It is employed for prediction-correction type estimation of unknown state variables^32^. In this study, the Kalman filter is utilized to estimate and forecast the trajectory of spatters, subsequently allowing for the calculation of particle velocity. The movements of the particles are considered as combination of uniform rectilinear motion with random disturbance. In the Kalman filter framework, the state vector of a specific target particle at time t is defined as ${{\varvec{s}}}_{{\varvec{t}}}={[{x}_{t},{y}_{t},{w}_{t},{h}_{t},{v}_{t,x},{v}_{t,y}]}^{{\top }}$, where ${x}_{t},{y}_{t}$ are the coordinates of the target particle's centroid, ${w}_{t},{h}_{t}$ are the width and height of the target particle's bounding rectangle, ${v}_{t,x},{v}_{t,y}$ are the velocities in the *x* , *y* directions, respectively. The observation vector is defined as ${{\varvec{z}}}_{{\varvec{t}}}={[{x}_{t},{y}_{t},{w}_{t},{h}_{t}]}^{{\top }}$. The prior estimate system state and posterior state at *t* are denoted by ${s}_{t}$ and ${\hat{s}}_{t}$ , respectively. ${{\varvec{P}}}_{{\varvec{t}}}$ and ${\hat{{\varvec{P}}}}_{{\varvec{t}}}$ represent the corresponding covariances. In this study, the state transition matrix ${\varvec{\Phi}}$ and observation matrix **H** in the Kalman filter are as follows:

2 $$\Phi =\left(\begin{array}{cccccc}1& 0& 0& 0& 1& 0\\ 0& 1& 0& 0& 0& 1\\ 0& 0& 1& 0& 0& 0\\ 0& 0& 0& 1& 0& 0\\ 0& 0& 0& 0& 1& 0\\ 0& 0& 0& 0& 0& 1\end{array}\right)$$

3 $$\text{H}=\left(\begin{array}{cccccc}1& 0& 0& 0& 0& 0\\ 0& 1& 0& 0& 0& 0\\ 0& 0& 1& 0& 0& 0\\ 0& 0& 0& 1& 0& 0\end{array}\right)$$

#### Prediction step

The calculation of the a priori estimate ${{\varvec{s}}}_{{\varvec{t}}}$ and covariance ${{\varvec{P}}}_{{\varvec{t}}}$ at time *t* can be expressed as:

4 $${s}_{t}=\Phi {s}_{t-1}+{w}$$

5 $${P}_{t}=\Phi {P}_{t-1}{\Phi }^{T}+\text{Q}$$

where $\boldsymbol{\Phi }$ is the state transition matrix, ***w*** is the process noise and ***Q*** is the process noise covariance. ${P}_{t}$ is the prediction of the movement of the particle.

#### Update step

The Kalman gain can be calculated as follows:

6 $${K}_{t}={P}_{t}{H}^{\text{T}}{(H{P}_{t}{H}^{\text{T}}+{R}_{t})}^{-1}$$

where H is the observation matrix and ${R}_{t}$ is the measurement noise covariance, and the calculation of the posteriori estimation and covariance is as follows:

7 $${\hat{s}}_{t}={\hat{s}}_{t}+{K}_{t}({z}_{t}-H{\hat{s}}_{t})$$

8 $${\hat{P}}_{t}=(I-{K}_{t}H){P}_{t}$$

#### Tracking method based on the Kalman filter

**Figure 5:**
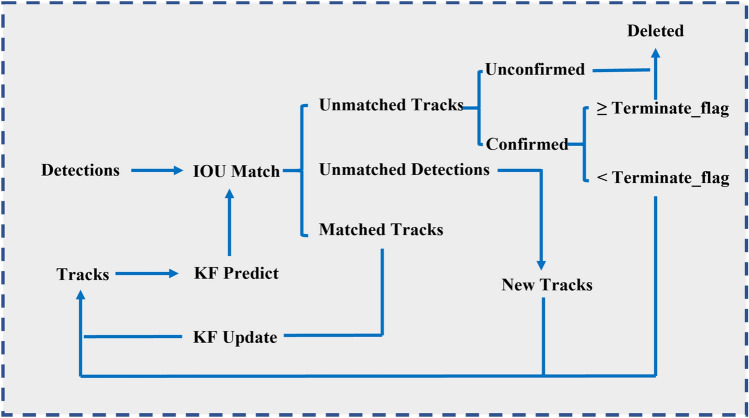
The schematic layout of the tracking method based on the Kalman filter.

**Figure 6:**
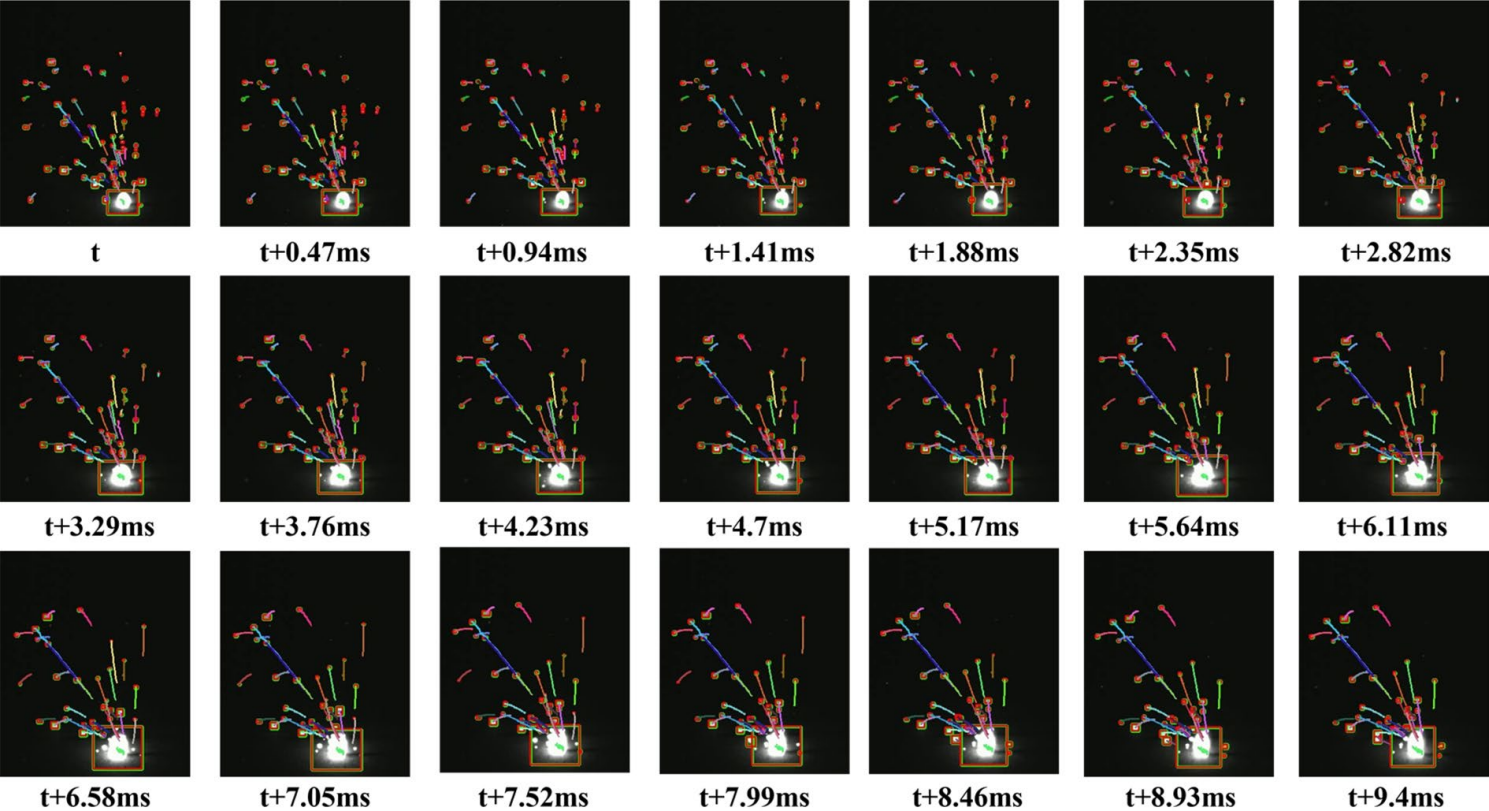
Schematic of the Kalman-filter-based spatter tracking method.

The comprehensive diagram of the tracking method is shown in Fig. 5. Within this diagram, "Detections" symbolize the detected target state information, while "Tracks" represent the trajectory details. Figure 6 shows the Schematic of the Kalman-filter-based spatter tracking method. The process of multi spatter tracking is shown in the Supplementary Video 1). The algorithm unfolds as follows:

1. For the first frame, create Tracks corresponding to the detected results. Initialize the motion variables of the Kalman filter and predict the corresponding bounding box through Kalman filtering. At this point, Tracks must be unconfirmed.
2. Match the detected bounding boxes of the current frame with the predicted bounding boxes from the previous frame based on the IOU (Intersection over Union), and find the optimal matching result using the maximum weight matching algorithm.
3. There are three possible outcomes: Unmatched Tracks, which will be deleted based on certain conditions; Unmatched Detections, which will be initialized as a new Tracks; and Successful matches, indicating that tracking was successful between the previous and current frames. Update the corresponding Tracks variables using the Kalman filter for these matches.
4. Repeat steps (2) and (3) until confirmed Tracks are obtained or the video frame ends.

### PSO-XGBoost algorithm

#### XGBoost algorithm

XGBoost is a gradient-boosting-based ensemble learning algorithm that combines excellent classification performance with fast execution speed^23^. For a dataset *P* containing *n* samples and *m* features, where *P* = {(*x*_*i*_, *y*_*i*_) | *x*_*i*_ $\in$ *R*_m_, *y*_*i*_ $\in$ *R*, *i* = 1*,*2*,* ⋯ , *n*}, *R*m and *R* represent the *m*-dimensional real vector dataset and the set of real numbers, respectively. The output value of the ensemble model is as follows:

9 $${\hat{y}}_{i}=\sum_{k=1}^{K} {f}_{k}({x}_{i}) , {f}_{k}({x}_{i})\in \text{F}$$

where *f* represents a regression tree, *K* is the total number of regression trees, and *F* represents the space of regression trees. The objective function is represented as follows:

10 $${O}_{bj}=\sum_{i=1}^{m} l({y}_{i},{\hat{y}}_{i})+\sum_{k=1}^{k} \Omega ({f}_{k})$$

where $l({y}_{i},{\hat{y}}_{i})$ is the loss function used to measure the error between the predicted classification value and the true value ${\hat{y}}_{i}$ is the predicted classification value, and ${y}_{i}$ represents the true value. $\Omega ({f}_{k})$ represents the regularization term. XGBoost algorithm adopts gradient boosting iterative computation. After each iteration, a new regression tree is added. Therefore, the result of the *t*th iteration is as follows:

11 $${{\hat{y}}_{i}}^{(t)}=\sum_{j=1}^{t} {f}_{k}({x}_{i})= {{\hat{y}}_{i}}^{(t-1)} + {f}_{t}({x}_{i})$$

and the objective function expression for the *t*th iteration is given by:

12 $${O}_{bj}^{(t)}=\sum_{i=1}^{m} l[{y}_{i},{\hat{y}}_{i}^{(t-1)}+{f}_{t}({x}_{i})]+\Omega ({f}_{k})+\sigma$$

To expand Eq. (4) using second-order Taylor approximation and incorporate the regularization term $\Omega ({f}_{k})$ to prevent overfitting, which can then be approximated as follows:

13 $$\begin{array}{cc}{O}_{bj}^{(t)}\cong & \sum_{i=1}^{m}[{\partial }_{{\hat{y}}_{i}(t-1)}l({y}_{i},{\hat{y}}_{i}^{(t-1)}){f}_{t}({x}_{i})+\\ & \frac{1}{2}{\partial }_{{\hat{y}}_{i}(t-1)}^{2}l({y}_{i},{\hat{y}}_{i}^{(t-1)}){f}_{t}^{2}({x}_{i})]+\Omega ({f}_{k})+\sigma \end{array}$$

14 $$\Omega ({f}_{k})=\gamma T+\frac{1}{2}{\lambda \parallel \omega }_{ }^{2}\parallel$$

where *γ* represents the leaf node penalty factor, *T* is the number of leaf nodes, *ω* represents leaf weights, and *λ* is the weight penalty factor.

#### Particle swarm optimization

Particle Swarm Optimization (PSO) is a swarm intelligence algorithm inspired by the foraging behavior of bird flocks. The PSO algorithm defines the solution to an optimization problem as the search for particles within a finite-dimensional space. Each particle is characterized by both a position vector and a velocity vector. All particles cooperate to find the optimal solution by continuously updating their positions based on their own best values and the best values found by the entire particle swarm. The fitness function evaluates the fitness of each particle's position, and all particles in the swarm follow the current best particle to explore the solution space.

Assuming a D-dimensional search space with a total of m particles in the swarm, the position of the i-th particle is represented as a vector *X*_*i*_ = [*x*_*i*1_, *x*_*i*2_, ⋯ , *x*_*iD*_], the velocity vector is represented as *V*_*i*_ = [*V*_*i*1_, *V*_*i*2_, ⋯ , *V*_*iD*_], and the best position found by the particle itself is represented as *P*_*i*_ = [*P*_*i*1_, *P*_*i*2_, ⋯ , *P*_*iD*_]. The overall best position found by the entire particle swarm is denoted as *P*_*g*_ = [*P*_*g*1_, *P*_*g*2_, ⋯ , *P*_*gD*_], where g represents the particle index, and *g* $\in$(1,2,3, ⋯ *m*). After initializing the particle swarm, the PSO algorithm calculates the fitness value for each particle and continuously updates and iterates to search for the optimal solution. In each iteration, the particle *X*_*i*_ updates its position and velocity based on its individual best value *P*_*i*_ and the swarm's best value *P*_*g*_. The iteration formula is as follows:

15 $${V}_{i}^{k+1} = \omega {V}_{i}^{k} + {c}_{1}{r}_{1}({P}_{i}^{k} - {X}_{i}^{k}) + {c}_{2}{r}_{2}({P}_{g}^{k}- {X}_{g}^{k})$$

16 $${X}_{i}^{k+1}={X}_{i}^{k}+{V}_{i}^{k+1} \left(i={1,2},\dots \dots m \right)$$

where *k* is the number of iterations; *ω* is the inertia factor, which is used to control the convergence and searching ability of the algorithm; *r*_1_ and *r*_2_ are random numbers between [0, 1]; *c*_1_ and *c*_2_ are the acceleration factors, which denote the weights of the acceleration terms that push the particles to the individual optimum *P*_*i*_ and the population optimum *P*_*g*_, respectively.

#### PSO-XGBoost model

**Figure 7:**
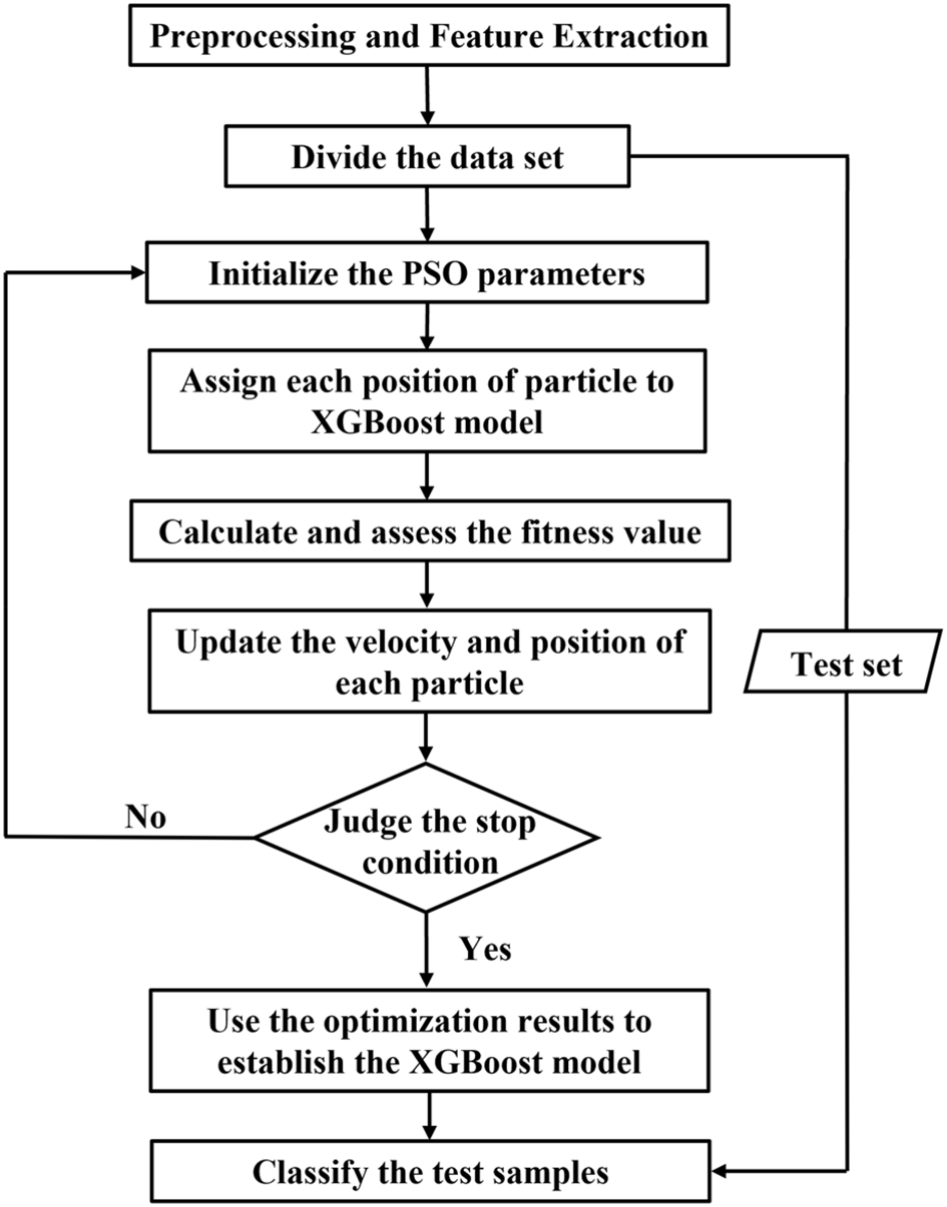
PSO-XGBoost algorithm flowchart.

Utilizing the principles of PSO and XGBoost theory, this study employs PSO to optimize the parameters of the XGBoost classifier, ultimately constructing a PSO-XGBoost classification prediction model. PSO-XGBoost algorithm flowchart is shown in Fig. 7. The specific procedure is shown as follows:

1. Data collection and preprocessing: in this experiment, a dataset comprising 5000 samples, each categorized into one of five classes, is collected.
2. Data splitting: we partition the data into a training set and a test set. Subsequently, we establish an appropriate fitness function and initialize the individual best values and global best value for the particles. This initialization process facilitates parameter optimization, including parameters like *learning_rate*, *max_depth*, *n_estimators*, and other parameters.
3. Particle velocity and position updates: we iteratively update the particle velocities and positions. Through the assessment of their fitness values, we continually refine the individual best values and global best value until the termination condition is satisfied.
4. Optimal parameter selection and model construction: finally, we select the optimal parameter values and employ them to construct an optimized XGBoost classification model. The training set is utilized to train this model.

## Results and discussion 

### Feature of spatters and plume

**Figure 8:**
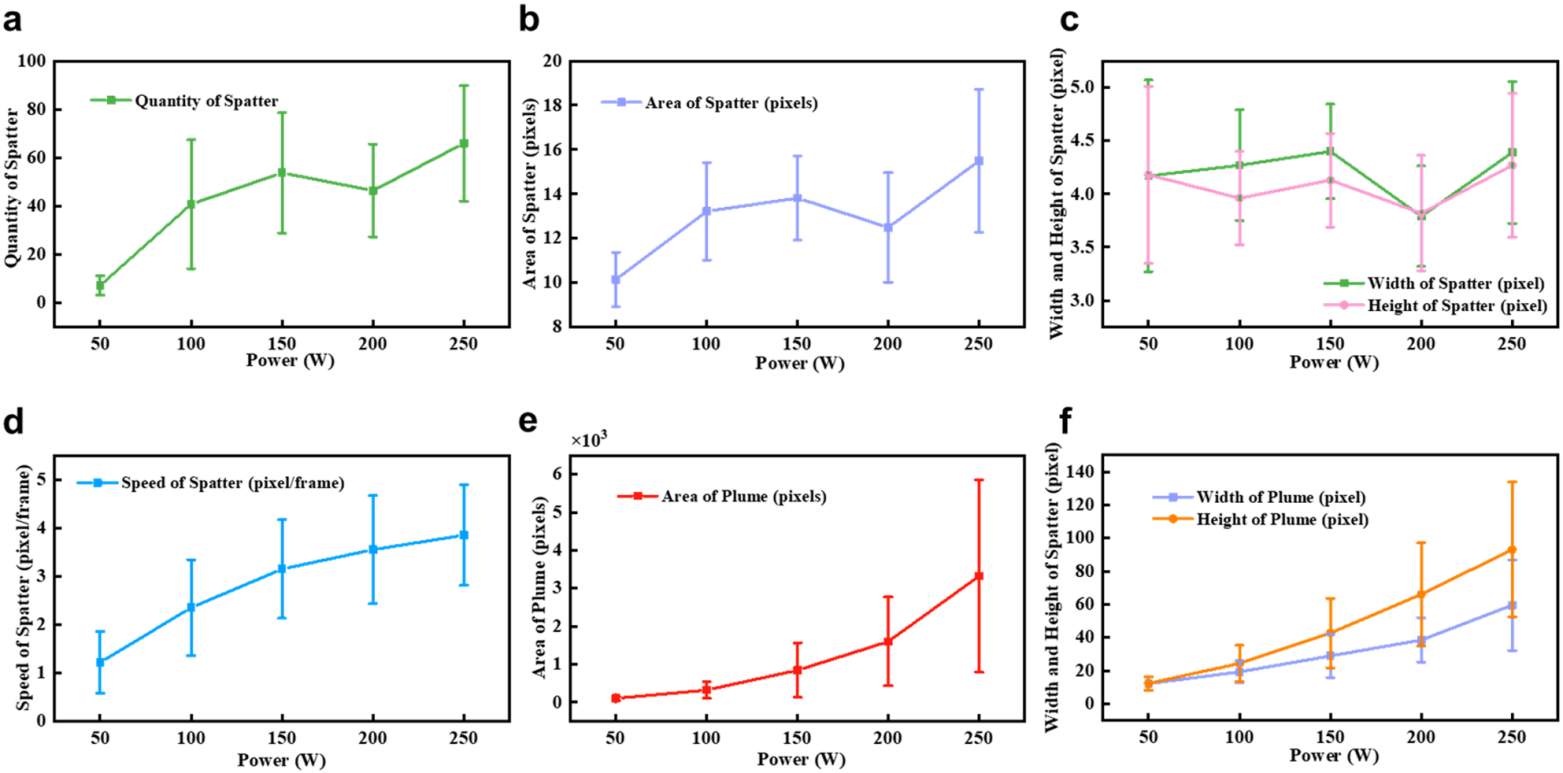
Analysis of plume and spatter features in different laser power when the laser scanning speed is 50 mm/s. **(a)** The average quantity of spatter. **(b)** The average pixel area of spatter. **(c)** The average pixel width and pixel height of spatter. **(d)** The average speed of spatter. **(e)** The average pixel area of plume. **(f)** The average pixel width and pixel height of plume.

**Figure 9:**
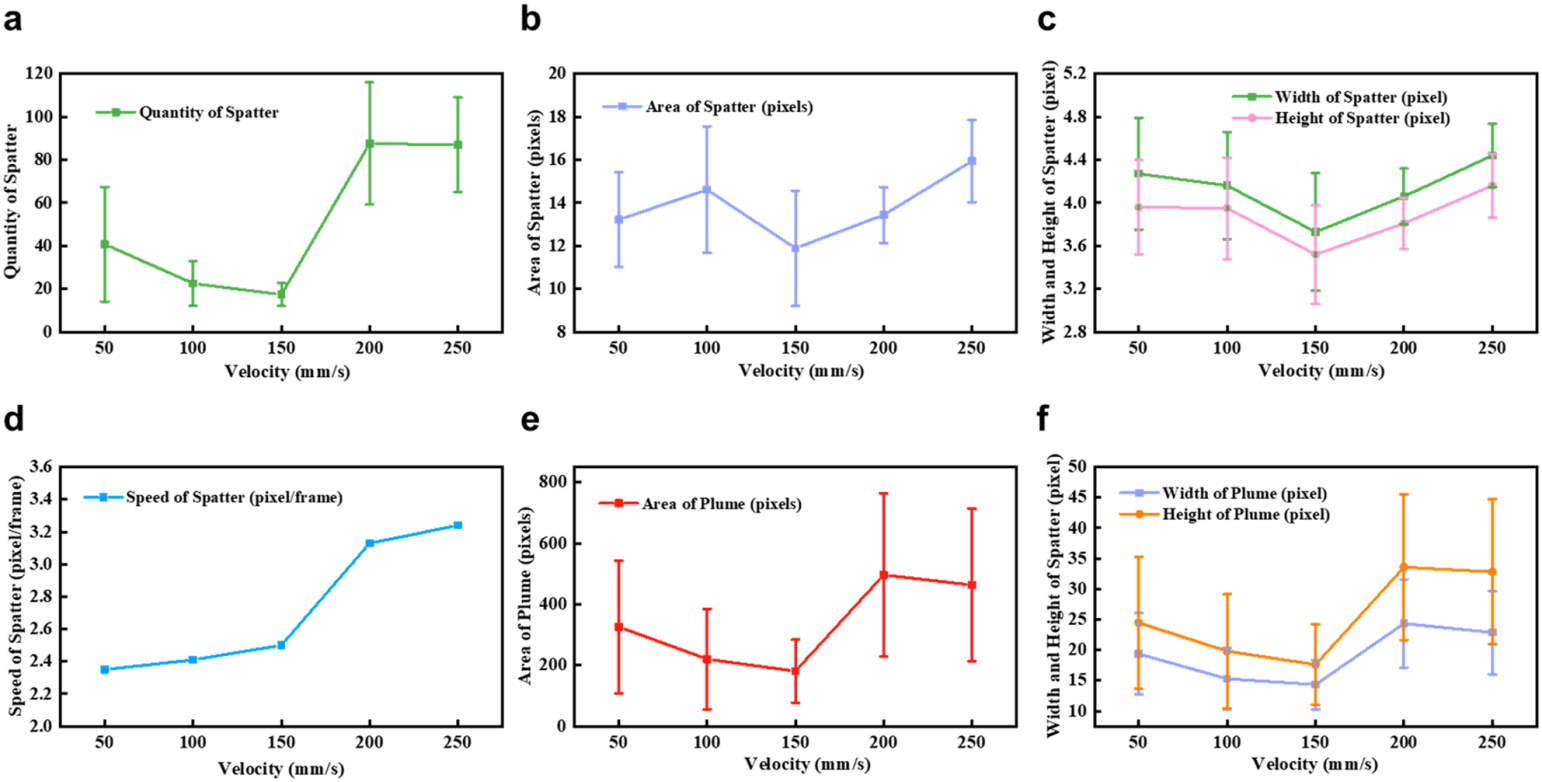
Analysis of plume and spatter features in different laser scanning speeds when the laser power is 100W. **(a)** The average quantity of spatter. **(b)** The average pixel area of spatter. **(c)** The average pixel width and pixel height of spatter. **(d)** The average speed of spatter. **(e)** The average pixel area of plume. **(f)** The average pixel width and pixel height of plume.

Figures 8 and 9 show the feature of spatters and plume. The average number of spatters refers to the average number of spatter particles per frame, while other features such as the average spatter velocity and average plume area refer to the average values per particle across all frames. With an increase in laser power, the interaction between the laser and material intensifies, magnifying thermal effects. Consequently, within the same duration, a more extensive melt zone emerges, producing a wider track and a pronounced influence on the powder. Additionally, the increase in laser power enhances the heat transfer effect, resulting in a wider and deeper affected region. The temperature fluctuations on the powder’s surface become more drastic, which generates a significant amount of metal vapor. As a result, the melt pool's stability diminishes, and as illustrated in Fig. 8a–c, both the quantity and size of the spatters escalate due to increased recoil pressure and surface tension.

However, when the laser power increases from 150 to 200 W, excessive energy input deeply melts the substrate, resulting in an increase in the viscosity of the melt pool and a decrease in the number and size of spatters, as shown in Fig. 8a–c. Although the formation of plumes becomes more prominent (Fig. 8e, f), the spatters are unable to separate from them, resulting in a diminished spatter count.

Between 200 and 250W, the mass of the molten material continues to increase, and the formation of liquid spatters gradually resumes. As the viscosity of the melt pool decreases, the size and quantity of spatters increase once again (Fig. 8a–c). Given the added energy and momentum, the plumes not only expand but the spatters also manage to detach from them, leading to a rise in their quantity and dimensions.

From an initial observation, as the laser scanning speed increases from 50 to 150 mm/s, both the quantity of spatters and the plume area decline (as seen in Fig. 9a, e, f). However, as the laser scanning speed continues to increase, there is a noticeable trend of an upward trajectory in the spatter parameters. This indicates that under the same laser power, a rise in scanning speed leads to a longer formed track length within the same timeframe, resulting in a larger region affected by the Bernoulli effect. Specifically, along the direction of laser travel, there are more powder particles on both sides of the weld bead being entrained by metal vapor, forming spatters. Consequently, the number of spatters increases with the improvement of scanning speed. This process may also involve spatter aggregation. Due to the relatively small overall change in input energy, the variation in spatter area is not significantly noticeable (Fig. 9b, c).

Additionally, as the power increases, the volume of evaporated material rises, leading to heightened recoil pressure. This subsequently results in an acceleration of spatter particle velocity, as depicted in Fig. 8d. However, there is no observed decrease in spatter velocity with an increase in laser scanning speed (Fig. 9d). This may be attributed to the fact that changes in the laser scanning speed at this juncture do not bring about a considerable shift in the input energy.

Notably, with parameters set to 50 mm/s-50W and 100 mm/s-100W, even though both maintain identical line energy densities, all process characteristics appear more accentuated in the latter, as illustrated in Figs. 8 and 9. This might be attributed to the simultaneous doubling of speed and power in the 100 mm/s-100W setting, directly correlating to a twofold increase in laser power density (i.e., power/beam area). Concurrently, this could lead to steeper temperature gradients and accelerated melting rates, potentially resulting in faster liquid metal movement and enhanced metal melting, thus impacting the melt pool's stability.

### Molten states

**Figure 10:**
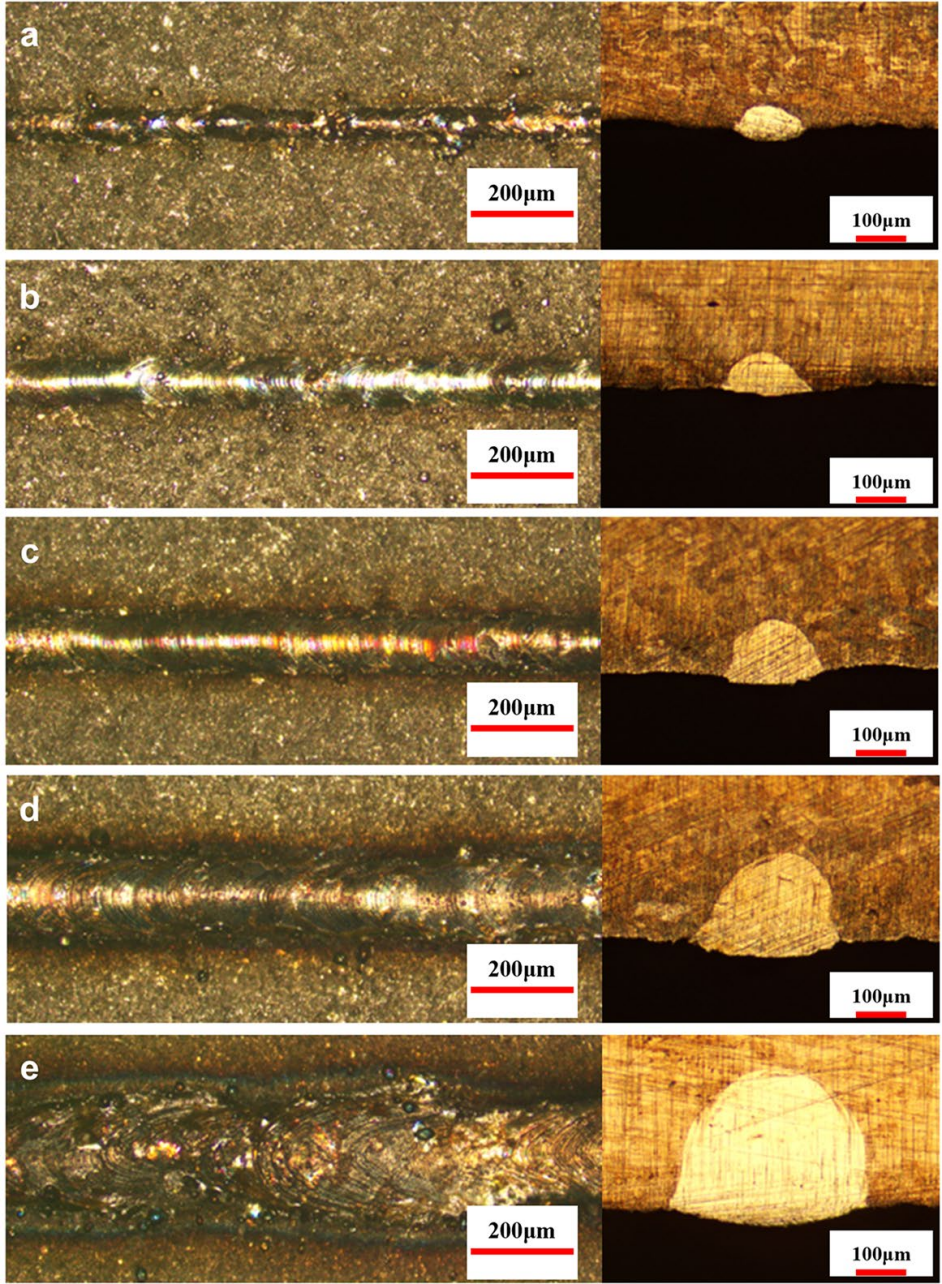
Molten states under different energy inputs. (**a**) Insufficient molten state. (**b**) Slight insufficient molten state. (**c**) Normal molten state. (**d**) Slight excessive molten state. (**e**) Excessive molten state.

**Figure 11:**
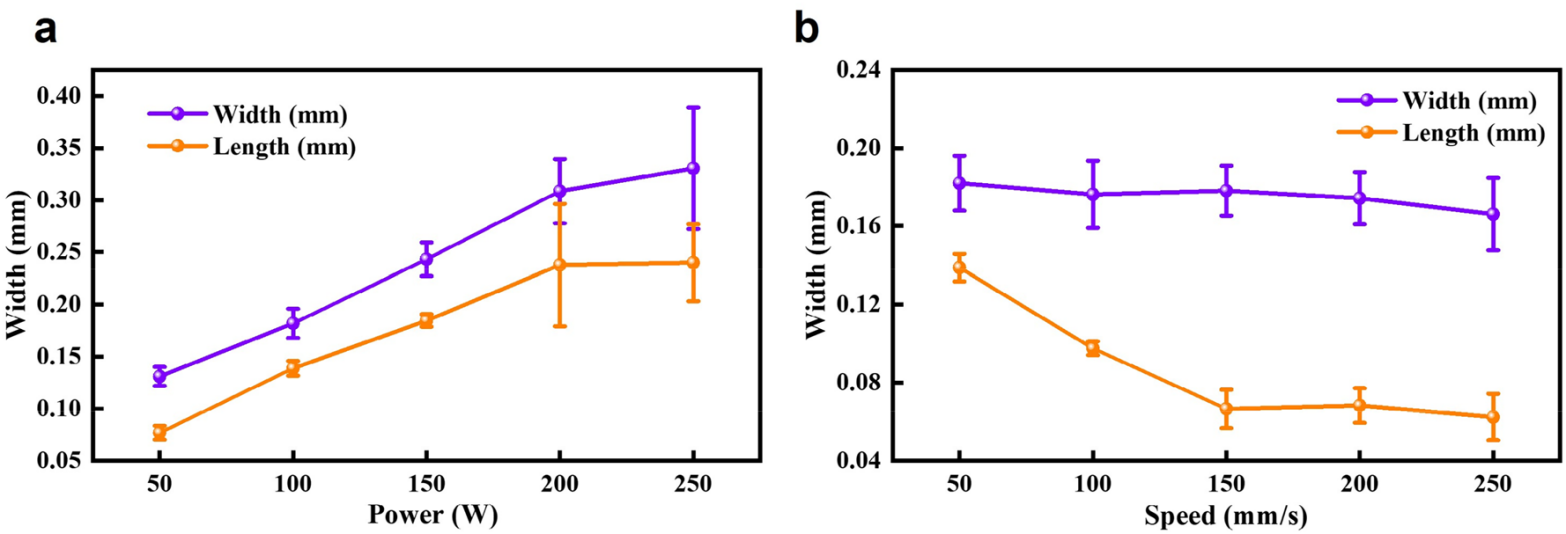
Analysis of melt track widths and lengths. (**a**) Changing with laser power. (**b**) Changing with laser scanning speed.

Depending on the morphology of the tracks at different energy inputs, it can be categorized into five different molten states: insufficient molten state (Fig. 10a), slight insufficient molten state (Fig. 10b), normal molten state (Fig. 10c), slight excessive molten state (Fig. 10d), and excessive molten state (Fig. 10e). From Fig. 10e, it can be observed that when the energy input is too high, the surface of the melt track is rough. Meanwhile, due to the large fluctuation of the melt pool, the consistency of width and height is poor. The track in the normal molten state is shown in Fig. 10c, with reduced track width, smooth and continuous surface, and good consistency of width and height. The undersupply molten state, as shown in Fig. 10a, has a smaller laser-affected area, resulting in a very thin melt track. At the same time, due to the low input energy, the wettability of the melt track is poor, and the continuity and consistency of width and height are poor, with obvious balling phenomenon. The slight excessive molten state, as shown in Fig. 10d, is between the excessive molten state and the normal molten state. And the slight insufficient molten state, as shown in Fig. 10b, is between the normal molten state and insufficient molten state. Moreover, as shown in Fig. 11, the width and height of the melt track increase with the increase of laser power, and decrease with the increase of laser scanning speed. In addition, the fusion channel shows a flat and wide characteristic, and its depth-to-width ratio is in the range of 0.35–0.80. This indicates that the high-energy laser beam shows significant diffusion and scattering of energy when acting on the metal powder.

### Results of classification

**Figure 12:**
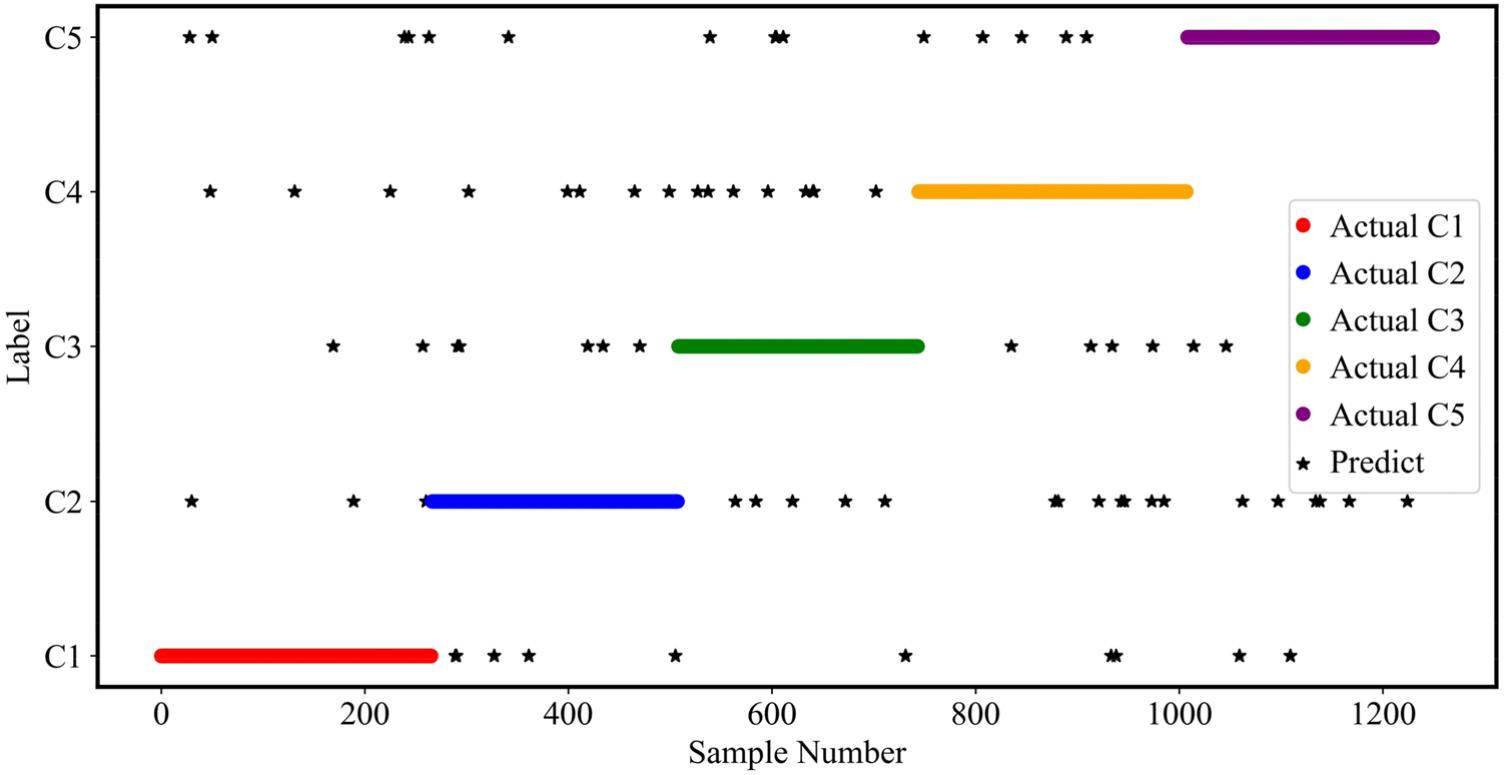
The prediction of the PSO-XGBoost classifier, where C1 to C5 represent insufficient molten state, slight insufficient molten state, normal molten state, slight excessive molten state and excessive molten state, respectively.

The relevant parameters were determined by the PSO algorithm respectively, and these parameters were used in the XGBoost. To train the proposed model, 75% of the data was randomly selected, and the remaining 25% was utilized to test and validate. K-fold cross-validation was selected for error estimation, and the classification accuracy was 92.16%. The predicted results of the model can be calculated by applying the model to the test set, as shown in Fig. 12.

**Figure 13:**
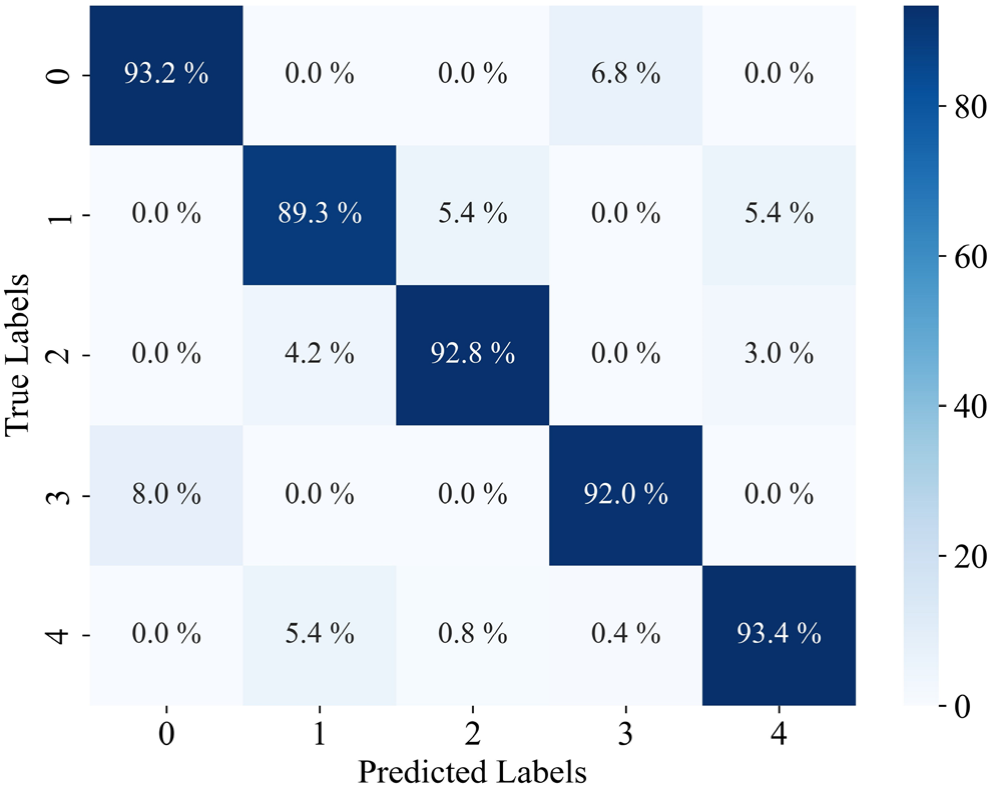
The confusion matrix of the PSO-XGBoost model.

The confusion matrix is a matrix where the actual categories form the rows and the predicted categories form the columns. It provides a visual representation of classification results. Each element in the confusion matrix represents a specific classification outcome. The diagonal elements of the matrix represent the number of correct predictions for each category. Therefore, a confusion matrix with larger values on the diagonal indicates better predictive performance. The confusion matrix of the test data is shown in Fig. 13. This indicates the feasibility of the proposed method to capture process characteristics and its potential as a tool for process monitoring.

**Figure 14:**
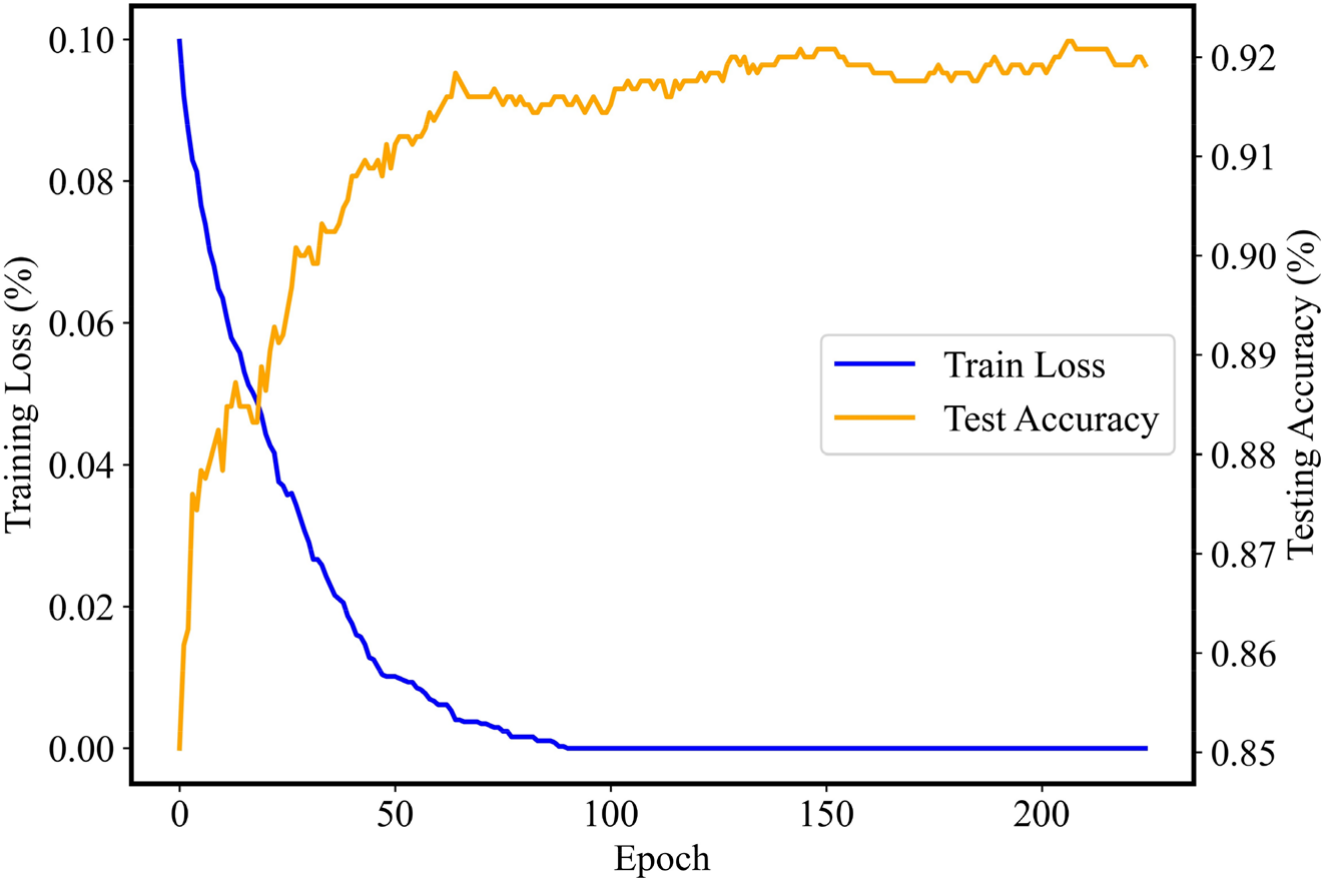
The training loss and accuracy plots of the PSO-XGBoost classifier.

The training loss and testing accuracy of the PSO-XGBoost classifier are also provided in Fig. 14. As observed in Fig. 14, the training loss of PSO-XGBoost classifier is close to zero after 90 epochs, and the testing accuracy is stable above 90%. It can also be concluded that the PSO-XGBoost classifier trained on the collected signals has high efficiency.

**Figure 15:**
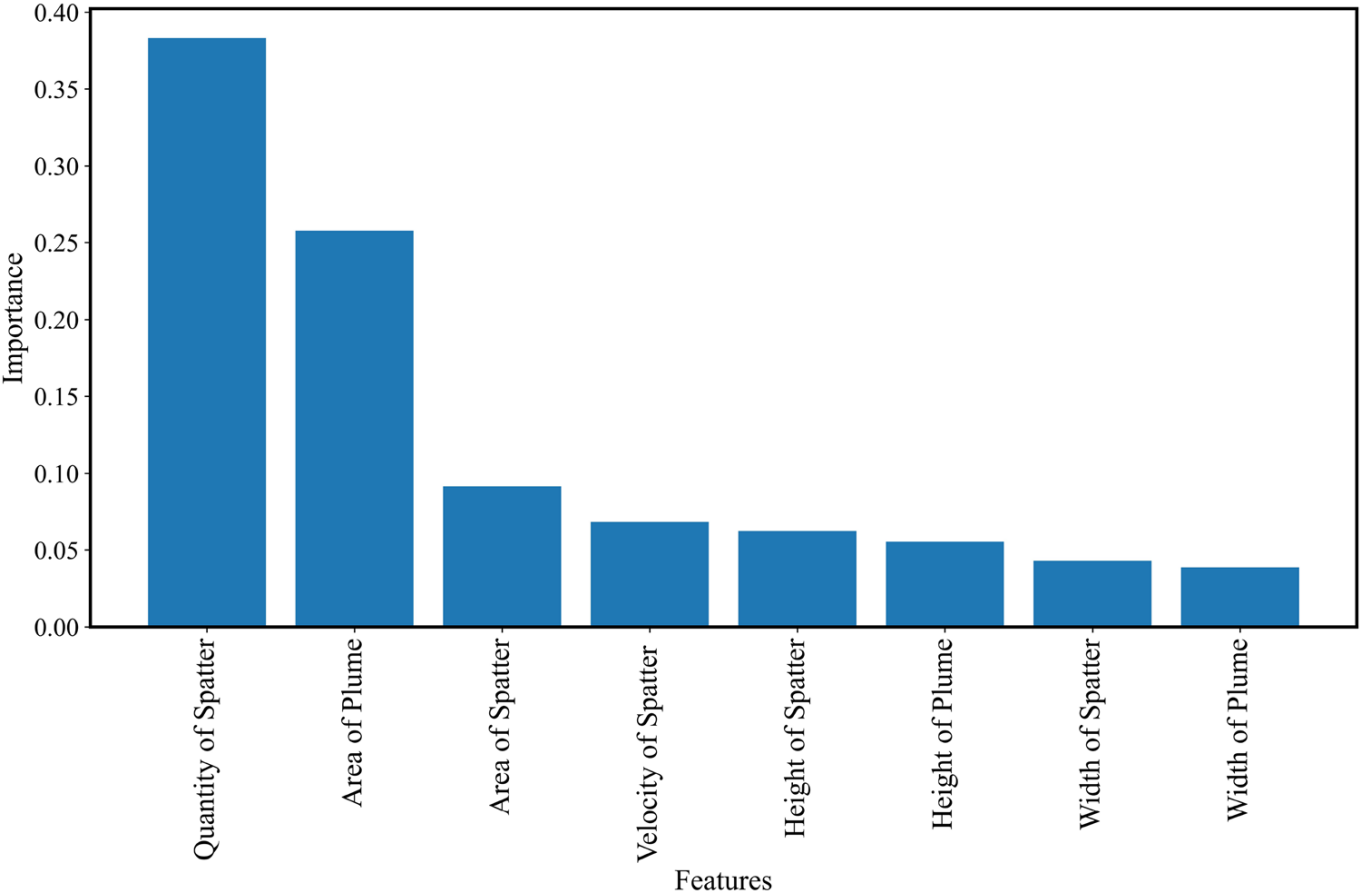
The importance of the features.

Figure 15 shows the importance of the features. Quantity of the Spatter, Area of Plume, Area of Spatter and Velocity of Spatter are the four features that are key variables when assessing the molten states.

**Figure 16:**
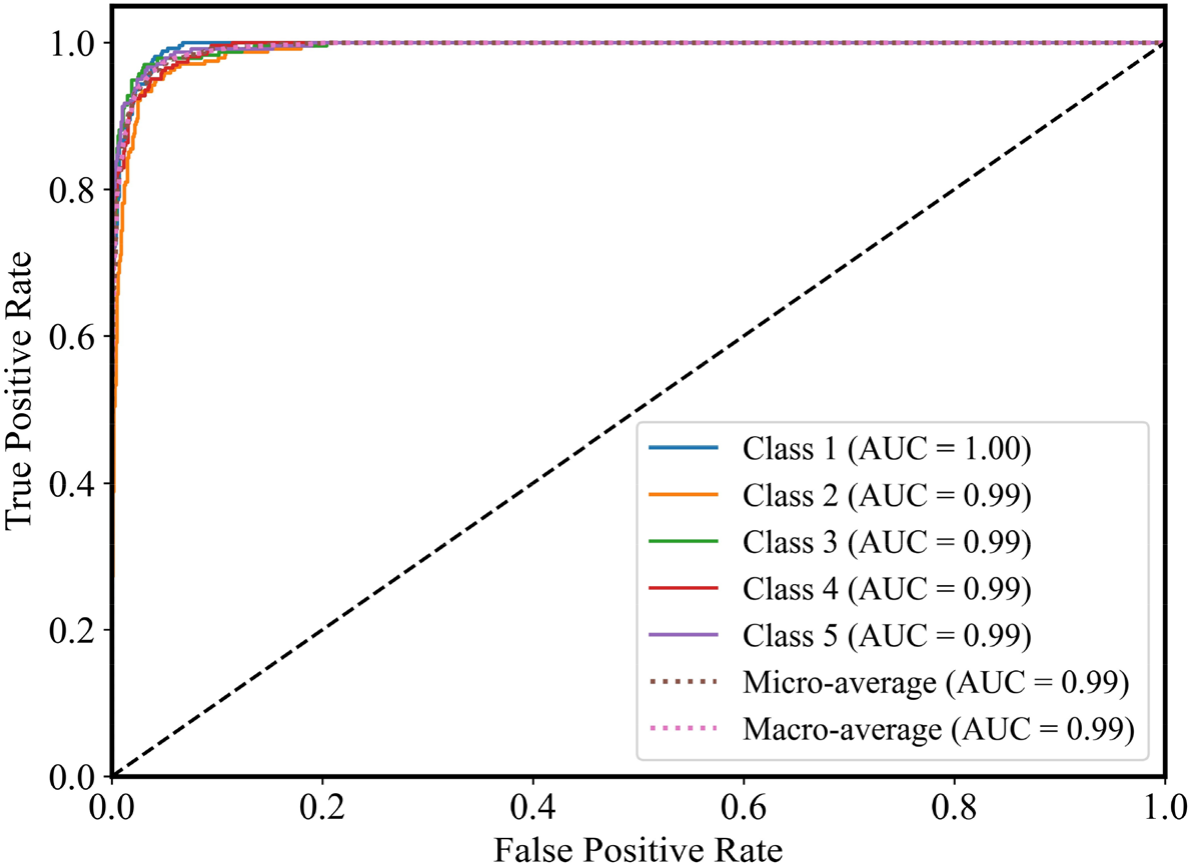
The ROC curves of all classes.

The ROC curves obtained from the PSO-XGBoost classification prediction model are shown in Fig. 16. Accordingly, it can be seen that the AUC values of the ROC curves for all categories are around 0.99, indicating that the model predicts well. In the multi-categorization scenario, the ROC curves are represented by macro average ROC as well as micro average ROC respectively. It can be seen that the micro average ROC and macro average ROC curves of the PSO-XGBoost classification prediction model both have an AUC value of 0.99, which is close to 1. This indicates that the model has a good classification performance, as well as reliability and accuracy.

The evaluation metrics of PSO-XGBoost and the other four models are shown in Table 3. Support Vector Machine's (SVM) evaluation metrics were observed to be the lowest among all models evaluated. This is potentially attributed to SVM's nature as a primarily linear classifier, which may struggle with capturing complex non-linear relationships inherent in certain datasets. Conversely, the PSO-XGBoost algorithm, by integrating PSO with XGBoost, introduces enhanced regularization techniques. These improvements not only boost the model's overall accuracy but also effectively mitigate overfitting issues, thereby demonstrating superior performance in handling both linear and nonlinear patterns present in the data. Compared to models such as SVM, RF, BP, and CNN, this mechanism in PSO-XGBoost is potentially more capable of maintaining the model's generalization ability when dealing with high-dimensional or noisy data. It can be observed that the proposed model has better performance. In comparison with algorithms like BP, CNN, RF, and SVM in LPBF process monitoring, PSO-XGBoost stood out by achieving higher accuracy and generalization due to its optimized hyperparameters, efficient handling of diverse features, computational efficiency, resilience to noise, interpretability via feature importance, and effective management of high-dimensional data, making it a superior choice that balances practicality with deep insights into the complex melting processes.

**Table 3:** The evaluations of different models.

Model	Accuracy (%)	Precision (%)	Recall (%)	F1-score (%)
PSO-XGBoost	92.16	92.16	92.14	92.15
BP	88.08	88.32	88.08	88.20
CNN	89.84	89.96	89.84	89.90
RF	89.04	89.41	89.04	89.22
SVM	80.4	81.43	80.4	80.91

## Conclusion

In this research, an off-axis vision monitoring system for Laser Powder Bed Fusion was developed using a high-speed camera. Different process parameters were set to obtain single tracks, and the printing process was recorded via the high-speed camera. In order to effectively extract the features of the process phenomenon, a method combined with the Otsu algorithm and Kalman filtering technique was proposed in this study. The area, width, and height of the plume, as well as the different characteristics of spatter such as area, quantity, velocity, width, and height, were studied in different scenarios. By monitoring the feature of spatters and plume in the LPBF process, we found that the linear laser energy density has a significant influence on the behavior of spatters and plume. The statistical data also showed that the width and height of the melt track were positively correlated with the energy input. From the measurements we found that the depth-to-width ratio of the melt channel was in the range of 0.35–0.80, which indicates the phenomenon of laser energy dilution. In addition, we found 5 states of the melt track including the insufficient molten state, the slight insufficient molten state, the normal molten state, the slight excessive molten state and the excessive molten state. In order to accurately evaluate the molten state, a molten state evaluation model based on PSO-XGBoost algorithm is proposed in this study. To overcome the difficulty of XGBoost algorithm parameter adjustment, PSO is used to optimize the parameters of XGBoost algorithm, which improves the efficiency and prediction accuracy. The experimental results show that the accuracy, precision, recall and F1 score of the PSO-XGBoost model are 92.16%, 92.16%, 92.14% and 92.15%, respectively, indicating that these eight types of characteristic information points of the spatters and plume have significant application value in reflecting the manufacturing status in the LPBF technology. Compared to other models mentioned in the paper, the obtained results offer enhanced interpretability and exhibit greater robustness and generalization capabilities. Overall, this research offers a novel and promising method for process monitoring in additive manufacturing.

In future research, we will investigate the effectiveness of machine learning in the challenging task of online monitoring of LPBF defects under complex and variable conditions. Improvements will be tailored to the different requirements of LPBF processes for defect monitoring under complex and variable conditions, including the development of a customized online monitoring software system.

### Supplementary Information

Supplementary Video 1.

## Data Availability

All of the data in this study are available from Yu Fu on reasonable request.
